# Adsorption of textile dyes from aqueous solutions onto clay: Kinetic modelling and equilibrium isotherm analysis

**DOI:** 10.3389/fchem.2023.1156457

**Published:** 2023-03-30

**Authors:** Metin Açıkyıldız, Ahmet Gürses, Kübra Güneş, Elif Şahin

**Affiliations:** ^1^ Advanced Technology Application and Research Center, Kilis 7 Aralık University, Kilis, Türkiye; ^2^ Department of Chemistry, K. K. Education Faculty, Atatürk University, Erzurum, Türkiye

**Keywords:** adsorption, textile dyes, clay, Langmuir isotherm model, pseudo-second order kinetic model

## Abstract

The commercial activated carbon commonly uses to reduce of dye amount in the textile industry effluents. In this study has focused on the use of a natural clay sample as low cost but potential adsorbent. For this purpose the adsorption of commercial textile dyes, Astrazon Red FBL and Astrazon Blue FGRL, onto clay was investigated. The physicochemical and topographic characteristics of natural clay sample were determined by scanning electron microscopy (SEM), X-Ray fluorescence spectrometry (XRF), X-Ray diffraction (XRD), thermogravimetric analysis (TGA), and cation exchange capacity measurements. It was determined that the major clay mineral was smectite with partial impurities. The effects of several operational parameters such as contact time, initial dye concentration, temperature, and adsorbent dosage on the adsorption process were evaluated. The adsorption kinetics was interpreted with pseudo-first order, pseudo-second order, and intra-particle diffusion models. The equilibrium adsorption data were analyzed using Langmuir, Freundlich, Redlich-Peterson, and Temkin isotherm models. It was determined that the adsorption equilibrium was reached in the first 60 min for each dye. The amount of adsorbed dyes onto clay decreased with increasing temperature, similarly, it decreased with increasing sorbent dosage. The kinetic data were well described by pseudo-second order kinetic model, and adsorption equilibrium data was followed both Langmuir and Redlich-Peterson models for each dyes. The adsorption enthalpy and entropy values were calculated as −10.7 kJ.mol^−1^ and −13.21 J.mol^−1^.K^−1^ for astrazon red and those for astrazon blue −11.65 kJ.mol^−1^ and 37.4 J.mol^−1^.K^−1^, respectively. The experimental results support that the physical interactions between clay particles and dye molecules have an important role for the spontaneous adsorption of textile dyes onto the clay. This study revealed that clay could effectively be used as an alternative adsorbent with high removal percentages of astrazon red and astrazon blue.

## 1 Introduction

Many industries such as plastic, leather, textile, food, drug, cosmetics, and paper produce an extensive quantity of the dye-containing effluents in their production processes while the Earth is trying to deal with serious problems such as climate change, global warming, greenhouse effect, loss of biodiversity, and overpopulation. It is known that over ten thousand different types of dyes and pigments are produced and 700-800 thousand tons of them are used annually in various industries especially in the textile industry around the world (Drumond Chequer et al., 2013; Hussaan et al., 2021). Large amount of water used in the pretreatment, dyeing, printing and finishing stages in the textile industries is polluted with dyes and released into the environment (Hussain and Wahab, 2018; Yaseen and Scholz, 2019). Textile wastewater has very different characteristics in terms of environmental issues such as dark color, wide pH range, high temperature, high chemical and biological oxygen demand (COD and BOD), high concentration of dissolved solids, metal ions and high conductivity (Wang et al., 2022).

The dyestuffs in textile wastewater are considered among the most important pollutants in the aquatic environment due to their complex structure, resistance to biological degradation, and serious effects on water quality, aquatic life, human health and ecosystem. Therefore, it is necessary to constantly monitor the concentration of these pollutants in wastewater and to implement effective and cost-effective treatment methods that ensure their removal from the effluent before it rises above critical values (Jawad and Abdulhameed, 2020; Dehmani et al., 2021; Loutfi et al., 2023). For this aim, the several physical, chemical, and biological methods were developed and used to treat of dye-containing wastewater such as adsorption (Kim et al., 2023), membrane filtration (Zhao et al., 2021), ion exchange (Joseph et al., 2020), photocatalytic degradation (Fu and Ren, 2020), coagulation-flocculation (Ihaddaden et al., 2022), electro-fenton (Jinisha et al., 2018), anodic oxidation (Mo et al., 2020), electrocoagulation (El-Ashtoukhy and Amin, 2010), advanced oxidation (Dadban Shahamat et al., 2022), reverse osmosis (Nataraj et al., 2009), enzyme (Riegas-Villalobos et al., 2020), bacteria (Srinivasan et al., 2022), yeast (Martorell et al., 2012), fungal (Gul et al., 2023), and algae (Zhang et al., 2022) assisted degradation (Al-Tohamy et al., 2022).

All of these methods have various advantages but they also have disadvantages such as high operating and maintenance costs, to cause secondary pollution with using large quantities of chemicals and producing sludge wastes (Gupta et al., 2001; Açıkyıldız et al., 2015). The adsorption process, which still maintains its value as an effective and well-known method in color removal from wastewaters, has superior properties compared to other methods in terms of high efficiency, low cost, low energy requirement, design flexibility, and ease of application (Rápó and Tonk, 2021; Gul et al., 2022). The commercial activated carbon is commonly utilized as an effective adsorbent in the adsorption process but researches are still carried out for organic or inorganic cheap and effective alternatives due to its high production and regeneration costs (Arellano-Cardenas et al., 2013; Abidi et al., 2015). Recently developed polymeric (Naciri et al., 2022) and metallic composites (Tanji et al., 2021) have been proposed as effective alternatives for the adsorption (Imgharn et al., 2022) or photocatalytic degradation (Fahoul et al., 2022) of metal ions (Imgharn et al., 2023), dyes (Hsini et al., 2021), and other organic pollutants. However, clay minerals have been mostly used to remove dyes from aqueous solutions (Bhattacharyya et al., 2014) since their properties such as non-toxicity, naturally abundancy, low-cost, high surface area, and porosity (Gürses et al., 2009). But, there are very few studies in the literature on the removal of astrazon red and astrazon blue, which are widely used in the textile industry, by adsorption on the clay surface.

This study aims to study the adsorption of two commercial textile dyes, astrazon red and astrazon blue, from aqueous environment onto natural clay. In addition, the study exhibits kinetic modeling and equilibrium isotherm analysis as well as the primary data on the effect of various experimental parameters such as initial dye concentration, sorption contact time, temperature, and sorbent dosage.

## 2 Experimental

The natural clay used as adsorbent was collected from the Kilis region in Turkey. The purification of the raw clay was carried out by decantation. The suspension was dried at 105°C for 24 h in an oven and then solid samples were milled and sieved to obtain a below 106 μm size fraction. The physicochemical and topographic properties of natural clay sample were determined by scanning electron microscopy (SEM), X-Ray fluorescence spectrometry (XRF), X-Ray diffraction (XRD), thermogravimetric analysis (TGA), and cation exchange capacity measurements.

**Figure 1:**
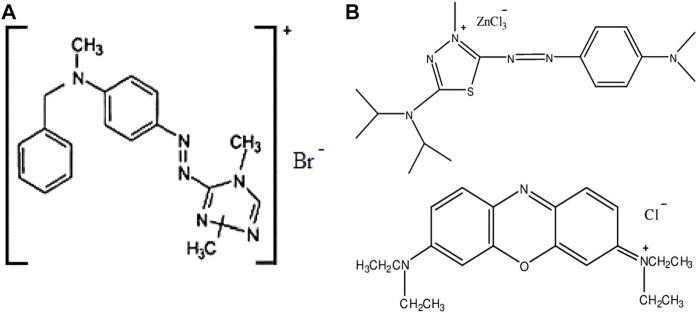
The chemical structures: Astrazon Red **(A)** and Astrazon Blue **(B)**.

As adsorbate commercial textile dyes, Astrazon Red FBL (AR) and Astrazon Blue FGRL (AB), were chosen. Astrazon Red FBL (C.I. Basic Red 46) is a cationic dyestuff and its formula is C_18_H_21_N_6_Br. Astrazon Blue FGRL is a cationic dye consists of two main components, which are C.I. Basic Blue 159 (C_17_H_27_Cl_3_N_6_SZn) and C.I. Basic Blue 3 (C_20_H_26_ClN_3_O). The ratio of the two components is 5:1 by weight, respectively. Though astrazon blue is a mixture of two basic components the following sections will consider it as a single dye species. The dyes were supplied from a textile factory in Gaziantep (Türkiye) and were of commercial quality. Their chemical structures are shown in Figure 1.

Adsorption experiments were carried out in 100 mL glass flasks immersed in a thermostatic shaker. Instead of comparing the adsorptive behavior of the two dyes under the same conditions, different experimental conditions were used for the two dyes, taking into account the findings obtained from the preliminary experiments, in order to meet the equilibrium conditions of dye adsorption for both dyes. A certain amount of clay sample (0.20 g for astrazon red and 0.15 g for astrazon blue) was added to 100 mL of the aqueous dye solutions of the different initial concentrations (20, 40, 60, 80, and 100 mg.L^−1^ for astrazon red, 25, 50, 75, 100, and 125 mg.L^−1^ for astrazon blue). The flasks with its contents were then shaken for the different adsorption times (15, 30, 60, 120, 240 min for astrazon red, 30, 60, 120, 180, and 240 min for astrazon blue) at 293, 313, and 333 K at constant stirring speed (130 min^−1^). At the end of adsorption period, the supernatant was centrifuged for 4 min at 3,500 min^−1^. The concentration of astrazon red and astrazon blue in the supernatant solution was calculated by using UV-Vis spectrophotometer (Biocrome Libra S70) at 531 and 601 nm, respectively. The adsorbed dye amount (mg.g^−1^) per gram of clay was calculated by considering the difference between the initial and final concentrations of the solutions.

## 3 Results and discussion

### 3.1 Adsorbent characterization

The chemical composition of the clay sample determined by X-ray fluorescence spectrometry (Rigaku RIX-3000) was presented in Table 1.

**TABLE 1:** Chemical composition of clay sample.

Constituent	%
SiO_2_	41.64
Al_2_O_3_	8.32
MgO	7.11
Fe_2_O_3_	5.83
CaO	4.06
Na_2_O	1.00
K_2_O	0.73
Loss of ignition	27.84

**Figure 2:**
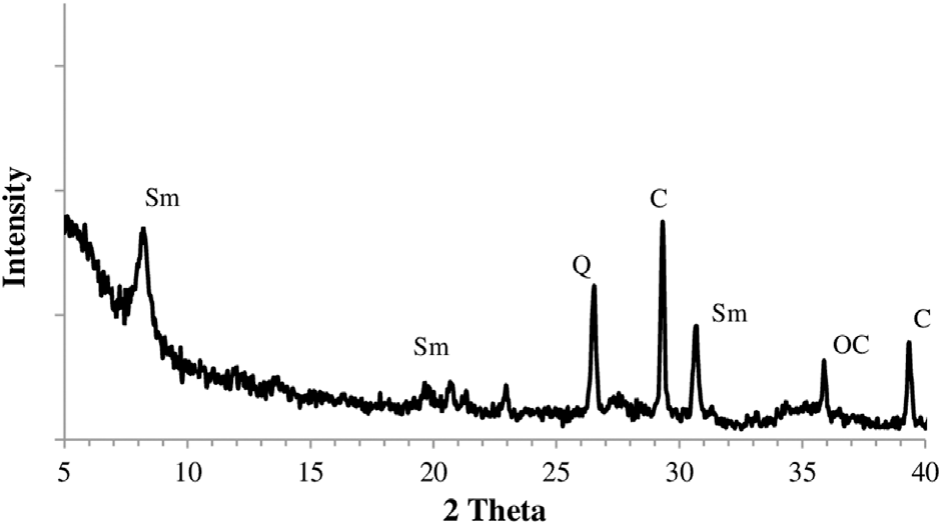
The X-Ray diffractogram of natural clay sample (Sm: Smektit, Q: Quartz, C: Calcite, OC: Opal-C).

The crystallographic analysis of the natural clay sample was performed using an X-ray Diffractometer (Philips X’Pert Pro) with a CuK_α_ (1.540 Å) radiation over a 2θ range of 2°–40° with 2°/min the scanning rate (Figure 2). It was determined from XRD results; the major clay mineral was smectite in the natural clay sample. In addition XRD patterns presented that natural clay sample still contained quartz, calcite, and opal-C as non-clay minerals (Önal and Sarıkaya, 2007). This is also consistent with the XRF findings presented in Table 1.

**Figure 3:**
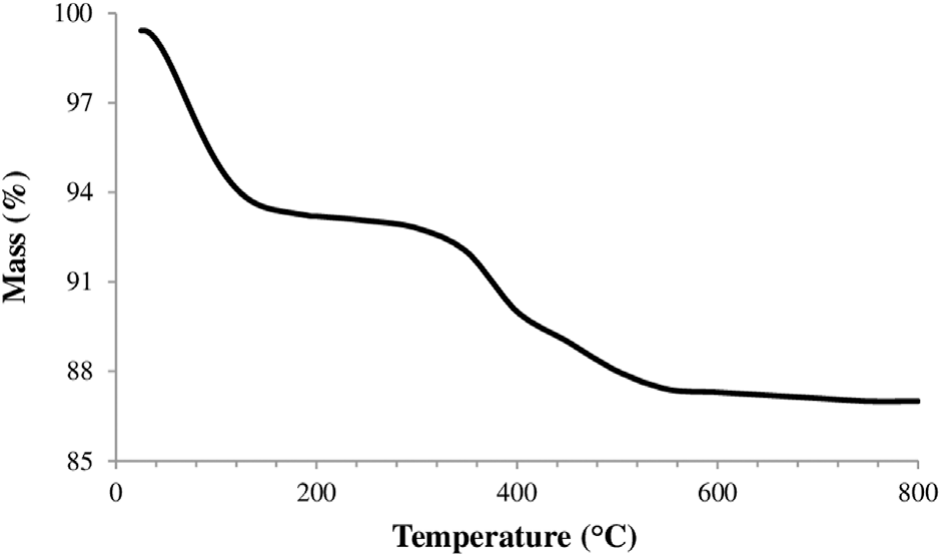
TGA thermogram of natural clay sample.

Thermogravimetric analysis (TGA) of clay sample was carried out using a thermogravimetric analyzer (Shimadzu DTG 60H) at a temperature range of 25°C–800°C and the obtained thermogram was presented in Figure 3. It can be seen from this figure the decomposition of natural clay evolved in two steps.

**Figure 4:**
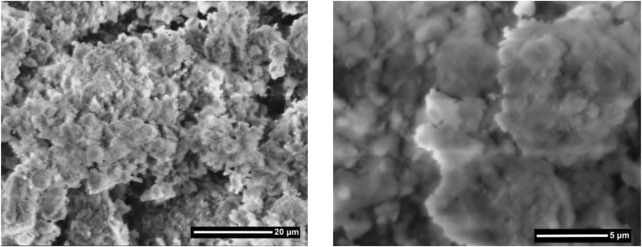
The SEM images of clay sample.

The first step is observed before 200°C with 6.2% mass loss. It is attributed to the dehydration of physically adsorbed water and water molecules around the exchangeable metal cations in the clay interlayers. The second step occurring with 4.8% mass loss over 350°C was attributed to the loss of dehydroxylation of the structural OH units of the clay (Önal and Sarıkaya, 2007). The topography and morphology of the clay sample was observed by FE-SEM (Zeiss, Supra 55) and the obtained SEM images were shown in Figure 4. The cation exchange capacity (CEC) of clay sample which determined the ammonium acetate method was 63 meq per 100 g clay (Gürses et al., 2009). The observed porous structure and the measured partially high cation exchange capacity offer the clay sample as a potential adsorbent.

### 3.2 The effect of initial dye concentration and contact time

**Figure 5:**
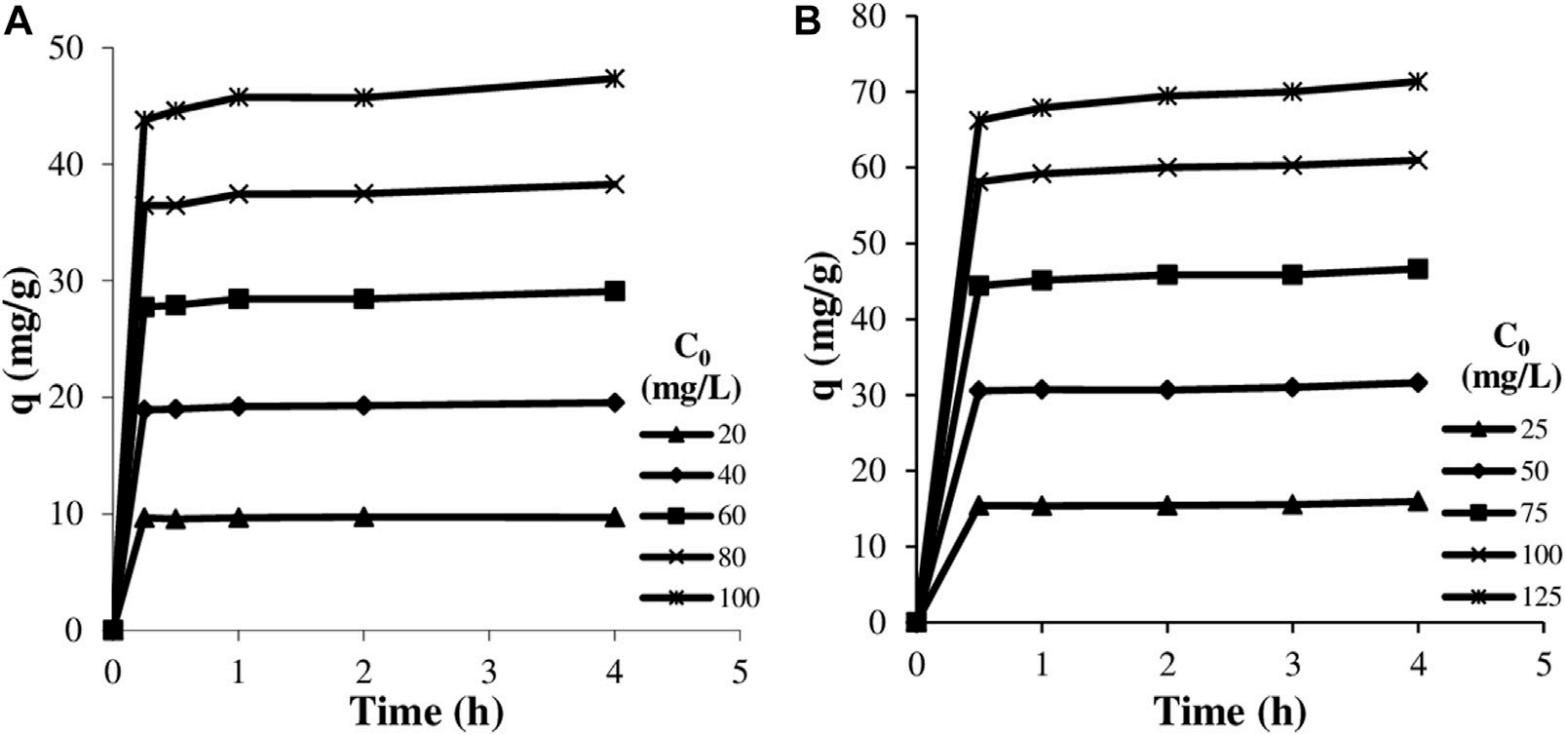
The variation of the amount adsorbed with adsorption time at various initial dye concentrations: astrazon red **(A)** and astrazon blue **(B)**.

The effect of initial concentration (20–100 mg.L^−1^ for astrazon red, 25–125 mg.L^−1^ for astrazon blue) on adsorption of the textile dyes onto clay at different times (15, 30, 60, 120, 240 min for astrazon red, 30, 60, 120, 180, and 240 min for astrazon blue) at 293 K was investigated. The results are given in Figures 5A, B.

As seen from these figures, while the initial dye concentration increased, the amount of adsorbed dye increased as expected. This situation can be explained by the concentration difference, which is the driving force in the adsorption of the dye from the solution to the clay surface. It is also seen that the amount of dye adsorbed for both dyes increases significantly up to 30–60 min and remains almost constant after this time. The high adsorption rate emphasizes the physical character of the adsorption process and evokes strong electrostatic interactions between cationic dye molecules and the negatively charged clay particles. However, due to partial differences especially at high concentrations, the adsorption equilibrium time was accepted as 60 min for subsequent experiments.

The fitting of the experimental kinetic data to the pseudo-first-order, pseudo-second-order, and intra-particle diffusion models was investigated in order to decide the adsorption mechanism of both dyes. The linear forms of pseudo-first order, pseudo-second order, and intra-particle diffusion model given in respectively below (Eqs 1–3) were used to calculate kinetic parameters:

$$\ln\left(q_{e}-q_{t}\right)=\ln q_{e}-k_{1}t$$ (1)

$$\frac{t}{q_{t}}=\frac{1}{k_{2}q_{e}^{2}}+\frac{t}{q_{e}}$$ (2)

$$q_{t}=k_{i}t^{1/2}+C$$ (3)

where, *q*_*t*_ is the amount of dye adsorbed at time *t* (mg.g^−1^), *q*_*e*_ is the adsorbed amount of dye at equilibrium, *k*_*1*_, *k*_*2*_, and *k*_*i*_ are the rate constants and *C* is a constant proportional to the boundary layer thickness (Gürses et al., 2006). The calculated kinetic values and fitting parameters for each kinetic model were presented in Table 2.

**TABLE 2:** Kinetic values calculated for adsorption of the textile dyes onto clay.

Dyes	C_init_ (mg.L^−1^)	Pseudo-first order			Pseudo-second order				Intra-particle diffusion		
		q_e,cal_ (mg.g^−1^)	k_1_x10^−3^ (min^−1^)	*R* ^2^	q_e,cal_ (mg.g^−1^)	q_e, exp_ (mg.g^−1^)	k_2_ (g.mg^−1^.min^−1^)	*R* ^2^	C	k_i_ x10^−2^ (mg.min^−1*/*2^.g^−1^)	*R* ^2^
Astrazon red	20	0.112	20.09	0.655	9.699	9.690	0.377	1.000	9.57	0.84	0.375
	40	0.674	8.71	0.902	19.569	19.538	0.049	1.000	18.724	5.29	0.967
	60	1.362	7.16	0.735	29.155	29.068	0.022	0.999	27.328	11.14	0.938
	80	1.971	8.58	0.764	38.462	38.259	0.016	0.999	35.829	15.85	0.919
	100	3.360	7.08	0.698	47.619	47.342	0.009	0.999	42.992	27.98	0.925
Astrazon blue	25	0.636	2.22	0.710	15.974	15.963	0.024	0.999	15.035	4.84	0.657
	50	1.205	3.36	0.813	31.646	31.622	0.014	0.999	29.925	9.30	0.768
	75	2.503	7.37	0.881	46.948	46.645	0.009	0.999	43.369	20.66	0.949
	100	3.440	9.58	0.973	61.349	60.978	0.008	0.999	56.893	26.61	0.966
	125	6.165	8.81	0.973	71.942	71.363	0.004	0.999	63.878	48.10	0.976

From Table 2, it can be seen that the experimental data have the highest fit to the second-order kinetic model. This very high agreement indicates that the adsorption takes place through strong electrostatic interactions, H-bonding and, n-π interactions. It can be said that the electrostatic interactions occur between the dye cations and the negatively charged surface, the H-bonding occurs between the H atoms on the clay surface and the N atoms in the dye molecules, and the n-π interactions occur between the lone pair electrons of the O atoms on the clay surface and the π orbitals in the aromatic dye chains (Gamoudi and Srasra, 2019; Jawad and Abdulhameed, 2020). In addition, the isotherm analysis results suggesting monolayer adsorption also supports this assessment. It can be seen that the rate constants calculated for astrazon red are higher than the rate constants calculated for astrazon blue when examining the data presented in Table 2 for the second-order kinetic model. This difference can be explained by the fact that relatively smaller astrazon red molecules diffuse faster from the solution to the adsorbent surface and then into the pores.

### 3.3 The effect of temperature

**Figure 6:**
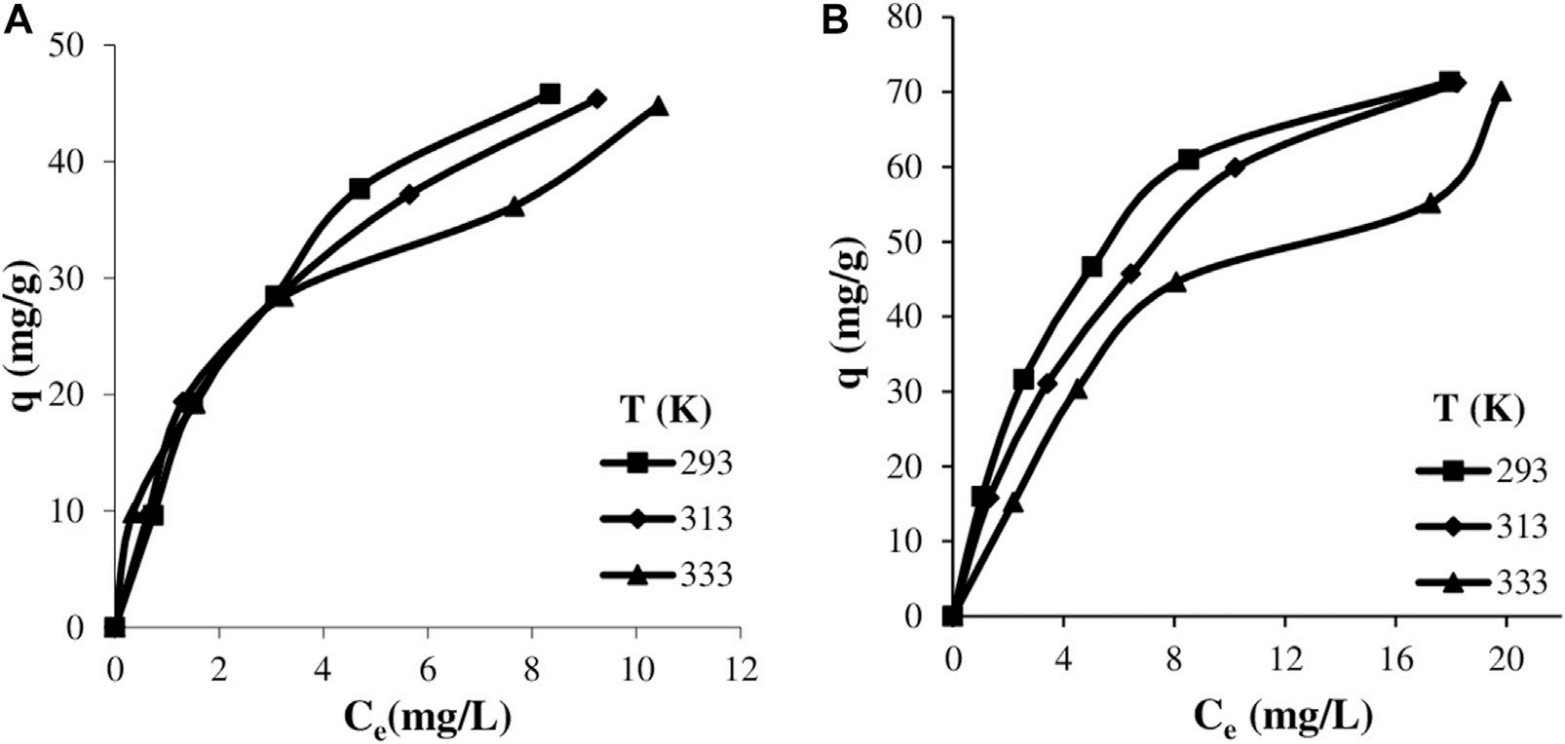
The variation of the amount adsorbed with equilibrium dye concentration for various temperatures: astrazon red **(A)** and astrazon blue **(B)**.

The effect of temperature on the adsorption of textile dyes onto clay was studied for the various initial dye concentrations at 293, 313, and 333 K. The experimental results are presented in Figures 6A, B, respectively.

It is seen from these figures that the isotherms are similar to the Type I isotherm especially at low and medium temperatures, that is, the amount of adsorbed dye increases significantly at low equilibrium concentrations, and then increases with lower slope as the equilibrium concentration increases, reflecting the formation of a plateau. It can be said that the isotherm obtained from the experiments carried out at 333 K is similar to the Type II isotherm, and after a plateau below the monolayer capacity formed at 293 and 313 K, the second layer begins to form at high equilibrium concentrations. When the isotherms are examined in general, it can be seen from Figures 6A, B that the adsorption process has an exothermic nature and the amount of adsorbed dye partially decreases as the temperature increases.

Thermodynamic parameters such as isosteric adsorption enthalpy (ΔH), isosteric adsorption entropy, and standard free energy change of adsorption were calculated by considering of the following equations (Doğar et al., 2010):

$$\frac{d\;\left(lnC\right)}{d\;\left(1/T\right)}=\frac{-\left(∆H_{ads}\right)}{R}$$ (4)

$$\frac{d\;\left(lnC\right)}{d\;\left(\ln T\right)}=\frac{∆S_{ads}}{R}$$ (5)

$$∆G_{ads}^{0}={∆H}_{ads}-T.{∆S}_{ads}$$ (6)

where, *C* is the equilibrium dye concentration in different temperatures, *T* is the absolute temperature, and R is gas constant (J.mol^−1^.K^−1^), respectively.

These calculations were based on the same amount of adsorbed dye at different temperatures corresponding to different equilibrium dye concentrations in which adsorption efficiencies were almost the highest (Doğar et al., 2010). The calculated thermodynamic parameters are given in Table 3. The calculated negative values of isosteric enthalpy and free energy changes for both dyes indicate that the adsorption process is spontaneous and exothermic (Brini et al., 2021). It can also be seen from this table that the calculated isosteric adsorption entropy changes for both ionic dye adsorptions are relatively small and of opposite sign. This can be related to the molecular sizes and degree of hydration of the dyes. It is expected that the adsorption entropy is negative as with astrazon red. In the adsorption of astrazon blue, which has more hydrophilic groups, it can be said that the increase in entropy becomes more dominant as a result of the increase in the degree of freedom of the hydrated water layers after the adsorption of the dye molecules. Depending on the change in Gibbs free energies, the tendency to be spontaneous of adsorption decreases for astrazon red and increases for astrazon blue while temperature increases. This is consistent with the exothermic adsorption process and the sign of entropy changes.

**TABLE 3:** The thermodynamic quantities calculated for adsorption systems.

Temperature (K)	Astrazon red			Astrazon blue		
	ΔG_ads_ (kJ.mol^−1^)	ΔH_ads_ (kJ.mol^−1^)	ΔS_ads_ (J.mol^−1^K^−1^)	ΔG_ads_ (kJ.mol^−1^)	ΔH_ads_ (kJ.mol^−1^)	ΔS_ads_ (J.mol^−1^K^−1^)
293	−6.83	−10.7	−13.21	−22.61	−11.65	37.40
313	−6.57			−23.36		
333	−6.30			−24.10		

### 3.4 Adsorption isotherms

In order to determine the mechanism of astrazon red and astrazon blue adsorption the experimental data were applied to Freundlich, Langmuir, Redlich-Peterson, and Temkin isotherm equations. The parameters and correlation coefficients calculated for the models are given in Table 4 together with the isotherm equations.

**TABLE 4:** Constant parameters and correlation coefficients calculated for various adsorption models.

Isotherm equations	Dye	Constant parameters		*R* ^2^
Freundlich	Astrazon Red	*n* = 0.610	k = 13.37	0.985
ln⁡q=ln⁡k+n⁡ln⁡C	Astrazon Blue	*n* = 0.537	k = 17.60	0.955
Langmuir	Astrazon Red	q_m_ = 67.11	K = 0.246	0.998
$\frac{C}{q}=\frac{1}{Kq_{m}}+\frac{1}{q_{m}}C$	Astrazon Blue	q_m_ = 90.91	K = 0.210	0.998
Redlich-Peterson	Astrazon Red	g = 0.986	B = 0.272	0.998
$\ln\left(A\frac{C_{e}}{q_{e}}-1\right)=g\ln\left(C_{e}\right)+\ln\left(B\right)$	Astrazon Blue	g = 0.982	B = 0.204	0.998
Temkin	Astrazon Red	K_T_ = 14.18	a_T_ = 2.66	0.989
$q_{e}=K_{T}\left(\ln a_{T}\right)+K_{T}\left(\ln C_{e}\right)$	Astrazon Blue	K_T_ = 20.30	a_T_ = 2.02	0.992

In these equations; C_e_ is the equilibrium dye concentration (mg.L^−1^), q_m_ is the maximum adsorption capacity (mg.g^−1^), q_e_ is the amount of adsorbed dye at equilibrium (mg.g^−1^), k is the Freundlich constant (adsorption intensity), 1/n is the order of adsorption, K is the Langmuir constant related to the energy or net enthalpy of adsorption, *A*, *B* and *g* are the Redlich-Peterson isotherm constants, *a*_*T*_ is Temkin isotherm equilibrium binding constant (L.g^−1^), *K*_*T*_ is constant related to heat of sorption (J.mol^−1^).

High agreement with the Langmuir and Redlich-Peterson (R-P) models observed for each dye is very important. The Redlich-Peterson (R-P) isotherm is a three-parameter empirical adsorption, both from Langmuir and Freundlich isotherms cover the relevant terms and can compensate for their existing shortcomings. Although the high fit with the Langmuir model implies that dye adsorption is limited to the monolayer, the observed fit with the Redlich-Peterson (R-P) isotherm also suggests that this coating is not necessarily ideal, and the homogeneity of the surface in terms of functional groups does not indicate that there is no lateral interaction between the adsorbed species. Accordingly, considering the layered structure of the clay, it can be claimed that both cationic dye ions are replaced by exchangeable cations in the interlayer region by the ion-exchange mechanism.

The high fit to the Langmuir model for each dye suggests that the clay surface is relatively homogeneous in terms of functional groups, dye adsorption onto clay is limited with a monolayer, and there is no significant interaction among adsorbed species. This high fitting also signs that the adsorption occurs predominantly through electrostatic interactions between dye cations and negatively charged clay surface (Gürses et al., 2004; Jawad and Abdulhameed, 2020).

The monolayer adsorption capacities determined from the Langmuir isotherm were compared with the maximum adsorption capacities reached in studies carried out under similar conditions for the removal of astrazon blue and astrazon red from aqueous solution (Table 5).

**TABLE 5:** The monolayer adsorption capacities of some adsorbents for astrazon blue and astrazon red adsorption.

Adsorbent	Adsorbate	q_m_ (mg.g^−1^)	Ref
Fungal (*Funalia trogii*) pellets	Astrazon red	42.24	Yesilada et al. (2002)
Coal mining wastes	Astrazon red	45.90	Almeida et al. (2010)
*Posidonia oceanica L.* leaves	Astrazon red	68.97	Cengiz et al. (2012)
Sepiolite	Astrazon red	106.0	Santos and Boaventura (2008)
Vermiculite	Astrazon red	44.00	Stawiński et al. (2017)
Acid treated vermiculite		64.00	
Base treated vermiculite		155.0	
Sepiolite	Astrazon blue	155.5	Karagozoglu et al. (2007)
Fly ash		128.2	
Activated carbon		181.5	
Chitosan/polypropenoic acid/ethylenediamine/magnetite nanocomposite hydrogel	Astrazon blue	194.1	Ali et al. (2022)
Macroalga (*Caulerpa lentillifera*)	Astrazon blue	30.67	Marungrueng and Pavasant (2006)
		80.70	Pimol et al. (2008)
Clay	Astrazon red	67.11	This study
	Astrazon blue	90.91	

When Table 5 is examined, it is seen that the monolayer adsorption capacities calculated for natural clay sample used in this study are above the average values for both dyes compared to other adsorbents used in the literature.

### 3.5 Effects of sorbent dosage

**Figure 7:**
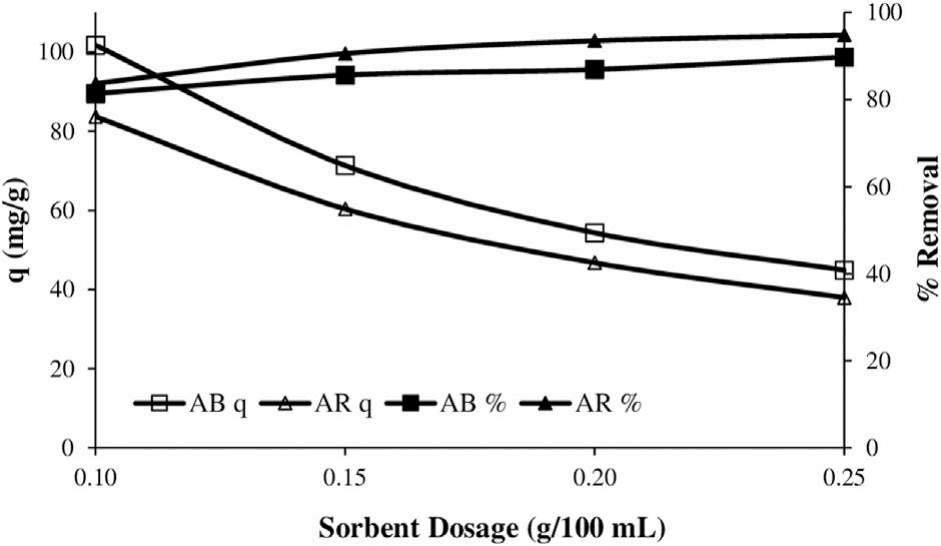
The variation of the amount adsorbed and the removal percentage with sorbent dosage.

The effect of adsorbent dosage was studied at 293 K, for 60 min, and initial dye concentration of 100 mg.L^−1^ and 125 mg.L^−1^ for astrazon red and astrazon blue respectively. The adsorbed amount of dyes decreased with increasing adsorbent dosage as expected. While dye removal (%) increased up 0.15 g and then it almost remains constant or slightly increased for each dye (see Figure 7).

The tendency of the adsorbed dye amounts to decrease against the adsorbent dosage can be explained that the increase in the amount of adsorbent does not mean that the active sites available for adsorption also increase proportionally, that higher amount of the adsorbent cause partial aggregation. In addition, this situation can be attributed that the unsaturation of the sites responsible for the adsorption process (Brini et al., 2021). The relatively increasing trend of removal ratios against adsorbent dosage also supports this claim. That is, although not proportional to the increase in amount, increasing adsorbent dosage led to a certain increase in dye uptake (Doğar et al., 2010).

## 4 Conclusion

In this study, the adsorption of cationic dyes Astrazon Red FBL and Astrazon Blue FGRL on clay was investigated. A purified natural clay sample which was characterized by XRD, XRF, and TGA analyzes has been used in the adsorption experiments. The effect of process parameters such as initial dye concentration, contact time, temperature, and solid/liquid ratio were examined for adsorption efficiency.

As a result of the findings obtained from these experiments:

■ When initial dye concentration increases, the amount of the adsorbed dye increases as expected,
■ The adsorption equilibrium is reached for both dyes in very low adsorption times. It was found that 60 min is sufficient in order to reach adsorption equilibrium for each dye.
■ The pseudo-second order model provided the best fit to the kinetic data among the investigated kinetic models.
■ The adsorption isotherms are similar to the Type I isotherm, especially at low and medium temperatures; at high temperatures, the isotherms in part suggest that the Type II isotherm and the second adsorbed layer begins to form especially at high equilibrium concentrations,
■ Equilibrium data fit well to Langmuir and Redlich-Peterson models for each dyes
■ The calculated Gibbs free energy and enthalpy changes are negative,
■ It has been determined that adsorption has exothermic nature and entropy decreases for Astrazon Red adsorption and entropy increases for Astrazon Blue adsorption.

As a result, it was determined that the natural clay sample could be used as an alternative adsorbent with a high removal ratio of 95% for Astrazon Red and 90% for Astrazon Blue.

## Data Availability

The raw data supporting the conclusion of this article will be made available by the authors, without undue reservation.
